# In Vitro Three-Dimensional (3D) Models for Melanoma Immunotherapy

**DOI:** 10.3390/cancers15245779

**Published:** 2023-12-09

**Authors:** Gemma Nomdedeu-Sancho, Anastasiya Gorkun, Naresh Mahajan, Kelsey Willson, Cecilia R. Schaaf, Konstantinos I. Votanopoulos, Anthony Atala, Shay Soker

**Affiliations:** 1 Wake Forest Institute for Regenerative Medicine, Wake Forest University School of Medicine, Winston Salem, NC 27101, USA; gnomdede@wakehealth.edu (G.N.-S.); agorkun@wakehealth.edu (A.G.); nmahajan@wakehealth.edu (N.M.); kwillson@wakehealth.edu (K.W.); cschaaf@wakehealth.edu (C.R.S.); kvotanop@wakehealth.edu (K.I.V.); aatala@wakehealth.edu (A.A.); 2 Wake Forest Organoid Research Center (WFORCE), Winston-Salem, NC 27101, USA; 3 Pathology Section, Comparative Medicine, Wake Forest University School of Medicine, Winston Salem, NC 27101, USA; 4 Department of Cancer Biology, Wake Forest University School of Medicine, Winston Salem, NC 27101, USA; 5 Department of Surgery, Division of Surgical Oncology, Wake Forest Baptist Health, Winston Salem, NC 27157, USA; 6 Medical Center Boulevard, Winston-Salem, NC 27157, USA

**Keywords:** melanoma, in vitro models, immunotherapy, tumor microenvironment, organoids, 3D bioprinting, microfluidics, tissue engineering

## Abstract

**Simple Summary:**

Melanoma treatment has progressed through the use of immune checkpoint inhibitors, significantly boosting patient survival rates. However, many tumors do not respond to these drugs or develop resistance, partly due to the tumor microenvironment influencing cancer cell growth and immune cell behavior. To gain insights into therapy outcomes, researchers have focused on developing cell-based ex vivo models of melanomas. These models include tumor organoids, engineered tissues, and microfluidic devices designed to replicate melanoma in its natural skin environment. Yet, integrating the tumor microenvironment and immune factors remains challenging. This review explores the creation of in vitro 3D models for normal skin and melanoma, focusing on methods to include immune components. These models hold promise for testing immunotherapies and uncovering resistance mechanisms. By faithfully replicating the tumor microenvironment and its interactions with the immune system, these models can enhance our understanding of immunotherapy resistance and ultimately improve personalized melanoma treatment.

**Abstract:**

Melanoma is responsible for the majority of skin cancer-related fatalities. Immune checkpoint inhibitor (ICI) treatments have revolutionized the management of the disease by significantly increasing patient survival rates. However, a considerable number of tumors treated with these drugs fail to respond or may develop resistance over time. Tumor growth and its response to therapies are critically influenced by the tumor microenvironment (TME); it directly supports cancer cell growth and influences the behavior of surrounding immune cells, which can become tumor-permissive, thereby rendering immunotherapies ineffective. Ex vivo modeling of melanomas and their response to treatment could significantly advance our understanding and predictions of therapy outcomes. Efforts have been directed toward developing reliable models that accurately mimic melanoma in its appropriate tissue environment, including tumor organoids, bioprinted tissue constructs, and microfluidic devices. However, incorporating and modeling the melanoma TME and immune component remains a significant challenge. Here, we review recent literature regarding the generation of in vitro 3D models of normal skin and melanoma and the approaches used to incorporate the immune compartment in such models. We discuss how these constructs could be combined and used to test immunotherapies and elucidate treatment resistance mechanisms. The development of 3D in vitro melanoma models that faithfully replicate the complexity of the TME and its interaction with the immune system will provide us with the technical tools to better understand ICI resistance and increase its efficacy, thereby improving personalized melanoma therapy.

## 1. Introduction

Melanoma is the most aggressive type of skin cancer. It develops from the malignant transformation of melanocytes, which are specialized pigment-synthesizing cells found mainly in the skin [1]. While melanoma accounts for only about 1% of all skin cancers, it leads to the most skin cancer-related deaths [2]. Early melanoma diagnosis is crucial for disease management. While the primary melanoma 5-year survival rate is 99%, this percentage declines to only 27% for metastatic melanoma [3,4]. 

The recent discovery and approval of immune checkpoint inhibitors (ICIs) for metastatic melanoma have transformed the therapeutic paradigm of the disease. Around 45% of patients respond positively to single-agent PD1 blockade [5], and 50% benefit from combination therapy with CTLA4 inhibition. These treatments increase the 5-year survival rate to 52% [6]. Still, a significant number of treated patients fail to respond, and some patients that initially respond later develop resistance. 

The tumor microenvironment (TME) plays a prominent role in the patient’s capability to respond to an immunotherapy treatment. The TME is a complex network of cellular and non-cellular components, including cancer-associated fibroblasts (CAFs), immune cells, endothelial cells, signaling molecules, and extracellular matrix (ECM) proteins, which surround the tumor cells and provide them with a highly supportive milieu. [7]. The interaction between cancer cells and TME has been shown to direct both tumor and TME development and progression, as well as tumor resistance to immunotherapies, likely due to the transformation of immune cells to a tumor-tolerant phenotype [8].

To properly study the crosstalk between the cancer cells and the TME, it is essential that we employ adequate models that recapitulate, as faithfully as possible, the characteristics of melanoma in its appropriate tissue environment. Both conventional 2D cell cultures and animal xenograft models fail to reproduce the complexity of the human TME and tissue architecture, which prevents their use to predict human drug responses accurately. However, during the past few years, there has been an effort to develop human cell-based 3D models that recreate these features, namely tumor spheroids, organoids, and tissue-engineered constructs.

One of the major challenges in the field remains the incorporation and modeling of the melanoma TME immune component. In previous studies, the immune compartment has been provided by either the heterogenic immune cell types within the tumor sample [9,10] or the addition of lymph node tissue from the same patient [11]. Nonetheless, these approaches overlook the role of the skin’s resident physiological immune cells in tumor mitigation. To overcome this hurdle, efforts made to generate physiologically accurate immune-competent 3D skin models [12,13,14] can serve as a base to incorporate melanoma cells and study the interaction between tumor, stroma, and the immune system.

This review aims to summarize the recent advancements in in vitro 3D melanoma modeling and the generation of immune-competent physiological skin models. By compiling this information, we intend to provide a framework for how these models could be integrated to achieve immuno-oncologically relevant constructs that can be further used to test and discover new immunotherapeutic agents and investigate resistance mechanisms.

## 2. In Vitro 3D Melanoma Models

Current in vitro 3D models of melanoma differ in the variety of cellular complexity and representation of tissue architecture. The 3D tumor modeling began with cancer spheroids. The use of these systems has been useful for modeling tumor complexity, heterogeneity, and clonality [15]. However, composed only of cancer cells, these spheroids fail to represent the intricacy of the TME, which contains other cell types and ECM molecules. As stated previously, the TME-tumor interaction is an integral determinant of cancer behavior and progression that can dictate response or resistance to immunotherapy treatment. In this section, we focus on in vitro 3D models of melanoma that represent at least part of the cellularity of the TME besides the tumor cells (excluding the immune component, which will be reviewed in the last section).

### 2.1. Melanoma Tumor Organoids

Multicellular tumor organoids are generated from freshly isolated tumor cells mixed with other cell types to mimic the pathophysiological parameters and characteristics of the in vivo solid tumors. Adding a specific cell type/s to the melanoma cells provides the opportunity to tailor studies that represent and assess specific TME features and their effect on tumor progression. For example, melanoma spheroids implanted into a fibroblast-laden collagen gel promoted the migration and infiltration of fibroblasts into the tumor. In turn, activated CAFs showed increased deposition of ECM proteins and growth factors, which provided the tumor with the chemical and structural support needed to become resistant to cisplatin treatment [16,17]. Thus, this model effectively reproduced the interaction between the tumor and the ECM and how this may impact susceptibility to chemotherapy.

Another hallmark of tumorigenesis is the formation of new blood vessels called angiogenesis. Additional blood supply can provide tumor cells with increased oxygen and nutrients, enhancing their growth, malignancy, and drug resistance [18]. Tumor cell-secreted angiogenic growth factors and endothelial cells are the main regulators of angiogenesis, and it is the crosstalk between tumor and endothelial cells that triggers the formation of new blood vessels. In fact, organoids assembled with either cell-line or patient-derived melanoma and endothelial cells (HUVECs) both showed the formation of capillary-like structures [19]. Nonetheless, each tumor type (i.e., cell line or patient-derived) interacted differently with the surrounding endothelial cells, which led to different angiogenesis degrees [19]. This model could be used to develop a personalized angiogenesis predictor model.

One of the limitations of multicellular melanoma organoids is that they fail to reproduce the architectural organization of the tissue, including the epidermal and dermal layers of the skin. To overcome this, Klicks et al. [20] generated a core of collagen IV and fibroblasts (dermal component) surrounded by a mixture of keratinocytes and melanoma cells (epidermal component). With this model, the authors described the role of melanoma cells in preventing keratinocyte maturation. They also revealed the existence of two distinct melanoma cell populations within the tumor: one invaded the dermal core, and the other one remained on the epidermal layer. Even though the epidermal melanoma population was mainly sensitive to docetaxel, some cells expressing high levels of the ABCB5 transporter were found to be resistant to the chemotherapeutic agent. Thus, not only this model could recapitulate the early stages of melanoma genesis, but it also pin-pointed the ABCB5 transporter as an interesting therapeutic target to prevent treatment resistance.

**Figure 1:**
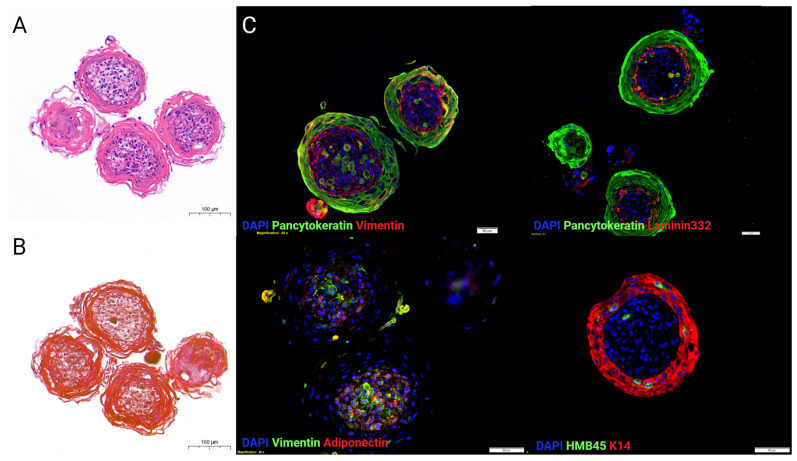
Skin organoids generated from primary skin cells. (**A**) Hematoxilin and Eosin (H&E) staining. (**B**) Masson’s Trichrome staining. (**C**) Immunostainings. Pancytokeratin and K14 antibodies were used to label the epidermal layer of the organoids, while Laminin332 delineated the keratinocyte basal layer. HMB45 stained neonatal melanocytes in the organoid. Vimentin and adiponectin antibodies were used to detect the fibroblasts and adipocytes in the stromal core that represent the dermis and hypodermis. Scale bar = 50um.

**Figure 2:**
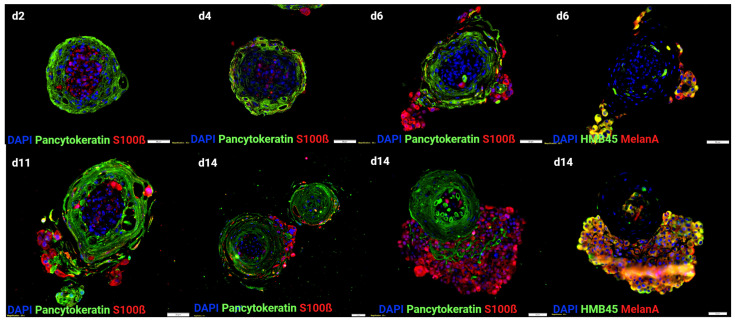
Skin organoids containing melanoma. Immunostainings. Pancytokeratin antibody was used to label the epidermal layer of the organoids, while SK-MEL-28 melanoma cells were positive for S100ß, HMB45, and MelanA antibodies. Throughout time, melanoma foci quickly grew and migrated outside of the skin organoid. Scale bar = 50um.

Despite being a useful tool for identifying, selecting, and optimizing drug candidates and therapeutic targets, the reported melanoma organoids do not represent the complete architectural features of the skin. Usually, in these models, keratinocytes do not fully differentiate and stratify due to the lack of specific signals from the underlying cells and non-physiological mechanical properties of the bioengineered skin. Overcoming this hurdle, our lab has successfully developed a spherical skin organoid model that represents the layered architecture of physiological skin, including a cornified epidermis containing mature keratinocytes and melanocytes and a dermal–hypodermal core composed of fibroblasts and adipocytes (Figure 1). We further added melanoma cells at the core of the skin organoids to simulate melanoma progression within the healthy skin tissue and study the interactions between healthy and cancerous cells. Such tumor organoid technology can also support high-throughput screening applications (Figure 2). Since they are spherical structures, these organoids do not simulate melanoma penetration through the skin layers and lack the ability to test the effects of melanoma spreading on the skin’s barrier function. Therefore, other approaches, such as tissue-engineered planar skin constructs, have been developed and are described in the next section.

### 2.2. Biofabrication of Human Planar Skin Constructs for Melanoma Research

Human planar skin constructs (hPSkCs) are generated from isolated primary human skin cells obtained from biopsies along with ECM components. These flat constructs are created layer-by-layer manually or using bioprinting technology and are subsequently cultured in an air–liquid interface to induce keratinocyte maturation and stratification [21]. Therefore, hPSkCs comprise a top layer of keratinocytes, a basement membrane, and a bottom layer of collagen-embedded fibroblasts, reproducing the epidermal and dermal components of the skin, making them a better model to study melanoma–skin interactions and tumor spreading. In these constructs, patient-derived melanoma cells can be incorporated in different ways. Co-seeding of melanoma cells with the keratinocyte layer allows for the modeling of the early stages of the disease to study melanoma genesis, spread, and progression [22,23]. Because the primary biopsy of melanoma is heterogeneous and contains different cell types, the addition of the melanoma cells into the hPSkCs as pre-assembled multicellular tumor organoids can standardize the contribution of each cell type to the model [24,25]. This approach would allow for controlling the number and size of melanoma organoids incorporated in the construct to simulate the different invasion patterns to the dermis or skin metastases.

One limitation of most hPSkCs is the use of non-human collagen matrices, which fail to recapitulate the appropriate skin microenvironment, especially the skin’s ECM architecture. To create a fully humanized ECM, Hill and colleagues [26] used a highly porous inert scaffold named Alvetex to support the growth of dermal fibroblasts and promote the deposition of their own ECM constituents, generating a stable human-like dermal compartment. Using this system, the authors seeded the melanoma cells onto the dermal equivalent and showed that melanoma cell invasion breaks down ECM collagens IV and VII, accurately reproducing the pattern of human melanoma invasion observed in vivo.

To increase model complexity, current efforts are directed toward generating skin constructs containing vascularization, appendages such as hair or sweat glands, and pigmentation [27]; however, the achievements reported to date are scarce. Recently, Bourland et al. [28] tissue-engineered a melanoma model containing both blood and lymphatic endothelial cells that mimicked angiogenesis and lymph vascularization, closely reproducing two key features of the TME. In this model, melanoma cells underwent apoptosis in response to a selective BRAF inhibitor, demonstrating the value of this system as a tool to test new therapies in a humanized environment.

Advancements in 3D bioprinting technologies could also be applied to create in vitro melanoma models. In 3D bioprinting, living cells and biomaterials are combined to enable the automated and standardized generation of specific tissue structures. To replicate the authentic skin tissue architecture using 3D bioprinting, it is important that the specialized bio-inks used to fabricate melanoma niches at distinct stages of the disease have a defined composition that ensures consistency and reproducibility [29].

### 2.3. Melanoma-on-a-Chip; Microfluidic Systems

Due to the lack of a robust integration of a perfusable vascular network, organoids, and planar melanoma–skin constructs do not fully simulate skin physiology and may fail to survive long-term experimentation. The combination of 3D cell culture and microfluidics may solve this problem. Microfluidics is a technology that enables precise delivery of fluids through channels measuring tens of micrometers. In what is called on-chip devices, including organ-on-chip and tumor-on-chip, the addition of living cells or organoids in a microfluidic system allows them to receive constant and controlled fluid flow and nutrient supply to maintain them, as the small interconnecting channels act as vessels that provide the cells or organoids with media. Besides flow, microfluidics can control many other parameters, including temperature, pH, oxygen supply, matrix structure and stiffness, and cellular composition and ratio [30]. This makes on-chip technologies more easily reproducible than other strategies described above. Ayuso et al. [31] employed this on-chip method and co-cultured melanoma with dermal keratinocytes and fibroblasts. The presence of stromal cells seemed to alter the morphology and growth dynamics of melanoma cells and their metabolic properties, thus underscoring the influence of the skin microenvironment on melanoma development and presenting new opportunities for therapeutic targets.

## 3. In Vitro 3D Models of Immune-Competent Skin

The skin contains inherent immune cells that act as the first barrier against an insult, like an infection or a tumor. These include tissue-resident CD8+ and CD4+ T cells and specialized immune cells. Langerhans cells (LCs) are found in the epidermis and serve as professional antigen-presenting cells. In the dermis, dermal dendritic cells (DCs) weakly stimulate T cells and instigate humoral immune mechanisms; macrophages can detect antigens entering through the skin and carry out an appropriate response, and skin-resident innate lymphocytes release cytokines and govern immunity and inflammation in the body surface [32]. Since the skin-resident immune system is likely to be the first line of the immune response to melanoma-specific antigens, it is important to include these immune cells in in vitro melanoma models. While no 3D model containing all skin immune cells has been established yet, this section summarizes the current attempts to model immune-competent skin for physiology or disease modeling.

### 3.1. Immune-Reactive Spherical Skin Constructs (Organoids)

To date, spherical human skin constructs, also called organoids, have been generated using stem cells as a starting point. The skin contains several populations of adult stem cells (ASCs) [33], which are activated after daily attrition, trauma, or disease to replenish the lost tissue. Numerous groups have described the generation of ASC-derived spherical skin constructs/organoids that model the epidermis, sweat glands, and hair follicles [34,35,36]. More recently, Lee et al. [37] reported on the generation of hiPSC-derived skin organoids capable of producing hair when implanted into mice. Despite the outstanding progress in this field, none of the generated organoids contained immune cells.

Incorporation of immune cells in organoids derived from other tissues can inform the approaches to generate immune-competent skin organoids. For example, microglia, the brain’s resident immune cells, have been successfully incorporated into brain organoids. Researchers parallelly generated brain organoids and hPSC-derived microglia. Then, at a time point chosen to represent microglial migration to the brain during development, they co-cultured the microglia and the brain organoids, generating immune-competent brain organoids [38]. Similarly, in intestinal organoids, there are numerous examples of organoid/resident immune cell co-cultures. In one study, Schreurs and colleagues [39] co-cultured patient-derived intestinal organoids with activated T cells to model the interaction between T cells and an inflamed intestine. Co-culture of Crohn’s disease patient-derived ileal organoids with autologous T cells served to observe the increased cytotoxic activity of mucosal T cells over epithelial cells in this condition [40]. Finally, microwell-based mouse intestinal organoids co-cultured with a murine macrophage cell line induced the loss of intestinal crypts in the organoids, therefore effectively modeling intestinal inflammation [41]. Thus, the parallel induction and co-seeding of organoids and immune cells could be utilized to incorporate the immune component into skin organoids.

### 3.2. Immune-Reactive Human Planar Skin Constructs (hPSkCs)

More significant advances have been made in the development of immune-competent 3D planar skin constructs, usually to model dermatopathological conditions. The simplest method to mimic immune responses in skin constructs is the addition of relevant cytokines in the culture. For example, Harvey et al. [42] added IL-22, IL-1α, IL-6, and TNFα to normal hPSkCs containing keratinocytes and fibroblasts, successfully mimicking a psoriatic phenotype in the epidermal layer. Similarly, Sriram and colleagues [43] treated hPSkCs with Th2 cytokines to model atopic dermatitis in vitro.

Other models have incorporated different types of immune cells to recapitulate the skin immune system’s reaction to external insults. To simulate the immune response after surface irritant allergen exposure, MUTZ-3 cells were differentiated into Langerhans cells (LCs) and co-seeded with the keratinocyte layer of an hPSkC onto a layer of collagen-embedded dermal fibroblasts. Researchers showed that similar to what occurs in physiological skin, LCs underwent a phenotypic shift to a macrophage-like state after irritant exposure while expressing epidermis-to-dermis migration markers [44].

A few studies have focused on the addition of CD4+ T cells in 3D skin constructs. Van den Boogard et al. [45] created hPSkCs with a decellularized, de-epidermized dermis encapsulating human primary keratinocytes and introduced allogeneic activated CD4+ T cells underneath. This resulted in keratinocyte activation, migration of T cells into the acellular dermis, and expression of psoriasis marker genes, thus reproducing a psoriatic phenotype. In another model for psoriasis, authors transferred hPSkCs containing primary dermal fibroblasts and epidermal keratinocytes onto CD4+ T cells polarized to Th1 and Th2. They demonstrated that these constructs exhibited a psoriatic epidermal phenotype that could be managed by adding hydrocortisone and anti-psoriatic drugs [13]. CD4+ T cells have also been used to model atopic dermatitis. Normal and filaggrin(FLG)-deficient (predisposing factor for atopic dermatitis) hPSkCs were cultured on top of naïve human CD4+ T cells. In this study, only FLG-deficient constructs exhibited higher levels of thymic stromal lymphopoietin (TSLP), which directly stimulated T cell migration and enhanced levels of pro-inflammatory cytokines, thus elucidating new knowledge on the pathogenesis of atopic dermatitis [12].

Lastly, macrophages have a crucial role in early antigen detection and wound healing, and they have also been studied in the context of healthy hPSkCs. Lègues and colleagues [46] bioprinted skin constructs containing primary keratinocytes, fibroblasts, and donor-matched CD14+ monocytes, which were differentiated into M1 and M2 macrophages. Upon activation with LPS and IL-4, the constructs exhibited an irritated dermis and damaged epidermis. In contrast, the presence of macrophages alone (not activated) did not increase the level of secreted cytokines, therefore making this system suitable for studying skin irritation and atopic dermatitis. In a different approach, Poblete-Jara et al. [47] inflicted a wound on a bioprinted hPSkCs composed of primary fibroblasts and keratinocytes. By adding a bio-ink containing macrophages and endothelial cells in the center of the wound, they could induce re-epithelization, thereby establishing a model to study macrophage-induced dermic wound healing.

As these models increase in complexity, one of the limitations encountered could be the use of miss-match donor cells in the same construct. Especially, including miss-matched CD8+ T cells and dendritic cells could give rise to unintended immune reactions within the model. This would hinder the correct evaluation of the immune response against tumor antigens. One of the strategies that could be employed to overcome this problem would be using autologous iPSCs differentiated into the different skin cell types. A human iPSC-derived hPSkC containing fibroblasts, keratinocytes, and melanocytes has been developed [48]; however, to date, there are no reports of iPSC models containing immune cells. A patient-specific immune-competent 3D skin model would also be relevant to assess personalized sensitivity to therapeutic agents.

### 3.3. Skin-on-Chip

Microfluidic skin systems have also been engineered to incorporate immune components. Similar to the case of hPSkCs, a common strategy to elicit an immune response in a microfluidic device is the treatment with cytokines. Wufuer and colleagues [49] constructed a three-layer device consisting of keratinocytes (epidermis), fibroblasts (dermis), and endothelial cells (endothelium). Each layer was connected by pores that allowed for intercellular communication. Treatment of fibroblasts with TNFα promoted the release of pro-inflammatory cytokines in the medium of endothelial cells, and this response could be abrogated with dexamethasone treatment. These findings established this system as a valuable model of skin inflammation that could be used to test the toxicity of cosmetics or drugs.

Other studies have approached the incorporation of immune cells in skin-on-chip in different ways. Ramadan and Ting [50] constructed an epidermal layer consisting of immortalized human keratinocytes over a layer of monocyte lymphoma cells (U937) that represented human DCs. When stimulated with LPS, U937 monocultures increased the expression of IL-6 and IL-1β; however, this effect was not as evident in the coculture with keratinocytes. Thus, this model suggested a novel role of the epidermal layer involving the regulation of immuno-reactive stimuli. Kwak et al. [51] engineered a skin chip containing epidermis, dermis, and endothelial layers. Neutrophil-like cells were added after skin construct maturation to reproduce leukocyte migration into the skin. Skin insults such as UV radiation or treatment with sodium dodecyl sulfate (SDS) increased the release of inflammatory cytokines and promoted leukocyte migration from the fluidic channels across the vascular layer and into the skin. A very similar approach was used by Ren et al. [14] to analyze the migration of T cells toward keratinocytes in inflammatory microenvironments. The injection of TNFα in the keratinocyte layer also promoted T cell transmigration across the endothelial layer and collagen gel towards the keratinocytes. Thus, both these models effectively mimicked the recruitment process of immune cells to inflammatory sites in response to an external stimulus. A comparable strategy could be used to simulate the recruitment of immune cells to the melanoma site.

Another approach is the perfusion of immune cells through skin organoid-containing chambers [52], which may require special design considerations. For example, it has been reported that specific types of ECMs used to generate organoids, such as MatrigelTM or Collagen I, may hinder the ability of immune cells to effectively migrate and infiltrate the organoids. A recent study showed that the embedding of gastric organoids in the synthetic matrix V-ORG-3 could sustain gastric organoid growth while also allowing the migration of flowing DCs into the epithelium, thereby reproducing the complex interactions between DCs and the gastric epithelium [53]. Thus, the exploration of new ECM materials could be crucial to building an accurate immune-competent skin-on-chip model.

## 4. In Vitro 3D Models of Immune-Competent Melanoma

The ability to correctly predict whether or not a patient will respond to immunotherapy is essential for implementing and improving treatment outcomes. In the TME, immune cells can play different roles. Cancerous cells, stromal cells, and the ECM in the TME can polarize immune cells to drive them to a growth-promoting action or tolerance state, thus interfering with their potential attack on the cancer cells. These mechanisms underlie the resistance of some patients to ICI therapy and tumor relapse [54]. To study the effects of melanoma on immune cells and vice versa, recent work has focused on adding or enhancing the immune compartment of melanoma 3D in vitro models. In this section, we summarize recent literature on the use of immune-competent melanoma models to evaluate immune responses, elucidate possible resistance mechanisms, and test immunotherapeutic agents.

### 4.1. Immune-Competent Melanoma Spherical Skin Organoids

Unlike the models discussed in the previous section, where immune cells were co-cultured with skin constructs, most of the immune-competent melanoma organoids described rely on the presence of endogenous immune cells from tumor biopsies. However, these studies have provided us with insights into the interactions between tumor cells and infiltrated immune cells within the TME. For instance, the culture of patient-derived melanoma organoids in an air–liquid interface has been shown to maintain the complex TME architecture, including stroma cells and tumor-specific tumor-infiltrated lymphocytes (TILs) [55]. These organoids effectively modeled immune response to ICI, such as anti-PD1 treatment, by inducing the expansion of CD8+ T cells within the organoid. Interestingly, this expansion was significantly higher in organoids from responder patients than in non-responders [55]. Therefore, this system could be potentially used for immunotherapy screening. Using a similar approach, a recent study elucidated a new mechanism of action of nivolumab (anti-PD1) in which the drug could drive off T-regs from CD8+ T-cells, avoiding their inactivation and, thus, enhancing their immunoactivity against the tumor [10].

Unresponsiveness to ICI has been previously related to T-cell peripheral tolerance. Yin et al. [56] generated melanoma organoids containing endogenous TILs and used nanoparticles containing innate immunity stimulants to maintain their activation, thereby preventing tolerance. They showed that these nanoparticles could increase the CD8+ T cell population and immunoactivity within the organoid at higher levels than anti-PD1 treatment, proposing their use in the clinic to activate TILs in patients who fail to respond to ICIs.

Instead of relying on endogenous tumor immune components, Votanopoulos and colleagues [11] incorporated immune cells into melanoma organoids by combining autologous lymph nodes and melanoma organoids in an ECM-based hydrogel system. They investigated the feasibility of using this method to predict individualized immunotherapy efficacy in the clinic. Even though the study’s sample size was modest, the results were very promising as the organoids’ response to various immunotherapeutic agents correlated with actual patient immunotherapy response in 87% of the cases.

When Troiani et al. [57] co-seeded patient-derived melanoma organoids with autologous lymphocytes in Matrigel^TM^, they found that tumor cells could affect the expression of the protein FKBP51s in immune cells. While upregulation of FKBP51s in T-regs was associated with a responder phenotype to anti-PD1 treatment, upregulation of the same protein in monocytes favored tumor-associated macrophage characteristics, which led to tumor tolerance and resistance to ICI. Therefore, this study elucidated FKBP51s expression in different immune populations as a new prognostic marker that could be used to select candidates for immunotherapy.

Despite the remarkable advances in generating immune-competent melanoma organoids, these models still fail to recapitulate the intrinsic skin tissue architecture in which the melanoma cells reside. Incorporation of melanoma and immune cells into a layered skin 3D model would allow us to evaluate tumor–microenvironment interactions more accurately.

### 4.2. In Vitro 3D Immune-Competent Human Planar Skin Constructs Containing Melanoma

The addition of immune cells to hPSkCs with melanoma has not been widely explored. However, a few articles have demonstrated its great potential in modeling the melanoma immune TME. A study found that melanoma cells incorporated in the dermal compartment of an hPSkC induce higher IL-10 release. When the supernatant of this construct was added to a monocyte culture, increased levels of IL-10 could drive monocyte differentiation to tumor-associated M2-like macrophages, thus describing a novel mechanism of immune tolerance in melanoma [23] and a possible therapeutic target to overcome it. The study was recently repeated, testing five additional melanoma cell lines. Each cell line recapitulated different melanoma stages. Nonetheless, only the cell line that mimicked a late invasive stage also produced increased cytokine expression. While the media conditioned by this construct could also drive the differentiation of M2 macrophages, the specific molecular factor responsible for this outcome remains to be determined [58].

Similarly, another work explored the effect of melanoma in modifying immune cell phenotype and tolerance. Researchers generated an hPSkC with primary fibroblasts and keratinocytes in which they introduced melanoma cells and a subset of PBMC-derived immunostimulatory DCs (named cDC2s). By interacting with the tumor cells, infiltrating cDC2s underwent a transformation into a distinct myeloid population that could no longer stimulate T-cell proliferation and immunoactivity. These findings suggested the relevance of this system for studying early tumoral immune-evasion events and the potential to use it to test ICI therapies in vitro [59].

Kaur et al. [60] investigated the effects of young and aged fibroblasts on melanoma progression and immune system interaction. To generate the skin construct, they embedded fibroblasts and melanoma in a collagen gel, to which they added autologous T cells. The study showed that young fibroblasts expressed higher levels of hyaluronan and proteoglycan link protein 1 (HAPLN1), which enhanced the migration and action of T-cells. This effect was abrogated when HAPLN1-deficient fibroblasts were used. Consequently, when aged constructs were treated with HAPLN1, fewer melanoma cells were detected, most likely due to the enhanced T-cell immunoactivity. Therefore, this model served to reveal a possible new therapeutic target to manage melanoma in older patients.

Still, one of the main limitations of these advanced models is the absence of blood vessels and lymphatics. This prevents the accurate mimicking of events such as angiogenesis and immune cell migration from the bloodstream to the tumor, which are crucial events in the TME. Once again, the use of microfluidic devices could offer a solution to this issue.

### 4.3. Immune-Competent Melanoma-on-a-Chip

To our knowledge, currently, there is not any microfluidic system containing healthy skin cells, melanoma, and immune cells together. However, some studies have investigated the interaction of melanoma and circulating immune cells using microfluidic devices. Because patient-derived melanoma spheroids retain autologous immune populations, they have been shown to respond to ICI treatment when integrated into a microfluidic culture and reproduce specific features associated with response and resistance to PD1-blockade, depending on the tumor’s origin (responder vs. non-responder patient) [9]. Thus, this culture method seems to be relevant for the evaluation of immunotherapy response and further development of precision immuno-oncology. A similar approach was used to determine the role of TANK-binding kinase 1 (TBK1) in tumor immune evasion. Inhibition of TBK1 in patient-derived spheroids placed in a microfluidic chamber enhanced the cytotoxic effects of the released cytokines in response to anti-PD1 blockade [61]; thus, this paper elucidated a novel potential therapeutic target to overcome resistance to immunotherapy.

Another aspect that can affect immunotherapeutic response is immune cell recruitment through the vascular system [62]. Using a microfluidic device representing the microvasculature, Ramos-Espinosa et al. [63] demonstrated that chitinase 3-like 1 (CHI3L1) altered the ECM and secretome of melanoma cells to promote immune cell recruitment and angiogenesis. It seems that CHI3L1 could be an interesting novel molecular target to modulate the patient’s anti-tumoral inflammation and response to therapy. In an akin microvasculature system, Chen et al. [64] showed that tumor cells can activate neutrophils and promote the production of extravasation factors that may disorganize the endothelial barrier and enhance the permeability of vessels and the probability of metastasis.

Although the discussed models lack the physiological skin component, they have provided insights into how cancer cells modulate immune cell recruitment, as well as testing response to immunotherapy. Combining these systems with other skin microfluidic devices described in the previous section may open the door to more physiologically accurate models that faithfully reproduce all aspects of the TME and can serve as platforms for therapy assessment and drug discovery.

## 5. Future Perspectives on the Clinical Applications of Melanoma In Vitro 3D Models

Cancer therapy continues to struggle with the lack of tools to accurately predict if an individual patient will have a favorable response to a specific treatment, usually prescribed based on cancer phenotype and not its molecular properties. As such, in vitro tumor models that best recapitulate the TME could help address this problem via the creation of patient-specific tumor organoids for drug response testing. However, maintaining a balance between the model’s complexity, reproducibility, and ease of use is the main challenge in creating a clinically applicable melanoma model in vitro. The most promising approach appears to include combinations of normal skin constructs that replicate skin microanatomy and physiology with particular subtypes of immune cells and patient-specific melanoma cells, all in a closed system like a microfluidic chamber.

Future-developed models could potentially be used to (1) identify novel therapeutic targets in cancer cells, (2) examine immunomodulation and ICI therapy, (3) find new early markers of carcinogenesis, and (4) improve patient-specific treatments. Further incorporation of new analytical methods, such as proteomics and spatial genomics, will replace conventional histology, allowing for a better understanding of the mechanisms driving normal cell recruitment into the TME, like cancer-associated fibroblasts.

## 6. Conclusions

The advent of ICIs for metastatic melanoma has brought a significant change in the way the disease is treated. Nonetheless, an ongoing challenge for this and other cancer therapies is the development of intrinsic and acquired resistance. The interplay between cancer cells and the TME can adversely affect the response to these therapies. Therefore, advancing our understanding of the interactions taking place inside the tumor niche will be crucial to overcoming the resistance hurdle. Due to the inability of 2D culture systems and animal models to faithfully recapitulate the cellular and tissue architecture complexity of the human skin and the melanoma TME, there is a need for more sophisticated cell-based ex vivo human melanoma models. More specifically, modeling the cross-talk between melanoma, healthy skin, and immune cells would greatly improve our capability to study and enhance the effects of immunotherapy as well as reduce time and cost for new drug development.

This review discussed different types of in vitro 3D models of cellular interactions within the skin melanoma microenvironment (Table 1). Each one of them differs in degrees of complexity, and as it increases, it is more physiologically similar to the in vivo conditions. Nonetheless, such complex models typically entail lower throughput and higher costs. Thus, it is important that researchers choose the system based on their specific needs. For example, organoids are prepared in fewer days and use fewer cells but have limited cellular complexity and less physiological and clinical predictive capabilities. hPSkCs and bioprinted constructs can effectively represent the tissue architecture in which melanoma and resident immune cells are found; however, the lack of perfusion prevents their long-term maintenance and modeling of immune cell recruitment from the vasculature. A microfluidic model may be better suited to reproduce later stages of melanoma and immune cell extravasation.

Besides the potential of these models to study melanoma-genesis and resistance mechanisms, the use of patient-derived cells to generate these constructs will also be relevant to advance precision medicine. Having these models readily available in the clinic will serve to choose the best treatment or select candidates for specific therapy.

As the field advances, there are still some gaps toward which more research should be directed. For example, there have not been any reports that attempted the generation of immune-competent skin organoids. Similarly, the formation of layered skin organoids incorporating melanoma cells remains to be fully described. Moreover, the use of microfluidic devices connecting melanoma compartments with other organoids could be explored as a model for distant metastasis.

In summary, the availability of in vitro 3D melanoma models that also mimic the heterogeneity of the tumor microenvironment and its relationship to the immune system will open the door to advanced studies to overcome ICI resistance, discover new drugs, and improve personalized therapy.

## Figures and Tables

**Table 1:** Overview of selected 3D in vitro models (organoids human planar skin constructs, and microfluidic devices) used to model different aspects of the skin physiology and architecture and the melanoma tumor microenvironment, with emphasis on the integration of the immune compartment.

Model	Method					Experimental Testing	Limitations	Reference
	Design	Cells			Support Matrix			
		Healthy	Cancer	Immune				
Spherical Melanoma Tumor Organoids	Co-culture with fibroblasts	Primary human skin fibroblasts (FF2441)	Human melanoma cell lines WM1366 or 1205Lu.	-	Bovine collagen I	Role of the stroma in tumor progression and therapy resistance.	Only one healthy skin cell type. No layered architecture.	[16]
	Co-culture with endothelial cells	Human umbilical vascular endothelial cells (HUVECs).	Human melanoma cell line A375. Melanoma cells derived from patient’s lymph node M21.	-	-	Tumor angiogenesis—crosstalk between melanoma cells and endothelial cells.	No healthy skin cells. Spheroid model is static so capillary networks are not functional. High variability depending on melanoma cells used.	[19]
	3 cell-type organoids	Human fibroblast cell line CCD-1137Sk Human keratinocyte cell line HaCaT	Human malignant melanoma cell line SK-MEL-28.	-	No ECM added. Collagen IV is produced by fibroblasts.	Early melanoma stages and response to chemotherapy.	Model lacks the formation of keratinocyte cornified layer.	[20]
	5 cell-type organoids	Primary fibroblasts, keratinocytes, melanocytes and adipocytes.	Human malignant melanoma cell line SK-MEL-28.	-	-	Melanomagenesis and tumor-stroma interactions.	Model does not simulate melanoma penetration through the skin layers	Unpublished data
Immune-competent spherical skin constructs (Organoids) and Melanoma	Air-liquid-interface	-	Heterogeneous stromal cells contained in the tumor biopsy.	Tumor-infiltrating lymphocytes, macrophages, B and NK cells from the biopsy tissue.	Type I collagen.	Personalized immunotherapy.	Model does not include healthy skin environment nor shows layered skin architecture.	[55]
	Air-liquid-interface	-	Melanoma biopsy. Heterogeneous population.	Immune cells from tissue biopsy.	-	Personalized immunotherapy.	Model does not include healthy skin environment nor shows layered skin architecture.	[10]
	Air-liquid-interface	-	Melanoma biopsy. Heterogeneous population.	Tumor-infiltrating lymphocytes	Type I collagen.	Immunotherapy.	Model does not include healthy skin environment nor shows layered skin architecture.	[56]
	Combined lymph node/melanoma organoids	-	Melanoma biopsy. Heterogeneous population.	Patient-matched lymph node.	Hyaluronic acid /collagen-based hydrogel	Personalized immunotherapy.	Modest number of patients. Model lacks healthy skin components and layered architecture.	[11]
	Co-culture with autologous lymphocytes	-	Melanoma biopsy. Heterogeneous population.	Autologous lymphocytes isolated from peripheral blood mononuclear cells	Matrigel.	Patient stratification and selection of candidates for immunotherapy.	Modest number of patients. Model lacks healthy skin components and layered architecture.	[57]
Melanoma on Planar Human Skin Constructs	Melanoma cells added to the cell mixture	Primary keratinocytes and fibroblasts.	Human melanoma cell lines WM35 and SK-MEL-28.	-	De-epidermised dermis (DED) prepared from skin tissue.	Melanoma progression and invasion to the dermis.	Model lacks interaction with other skin cell types such as melanocytes or adipocytes.	[22]
	Melanoma cells added as spheroids.	Primary keratinocytes and fibroblasts.	Human melanoma cell lines SBCL2, WM-115, and 451-LU.	-	Rat Tail Collagen I.	Drug testing for metastatic melanoma.	Model lacks other TME components such as endothelial cells that could modify tumor progression.	[25]
	Melanoma cells added between the dermal and epidermal layers.	Primary keratinocytes and fibroblasts.	Human melanoma cell lines WM35 and SK-MEL-28.	-	Alvetex scaffold, which promotes ECM deposition by fibroblasts.	Melanoma progression and invasion.	Model lacks other TME components such as endothelial cells that could modify tumor progression.	[26]
	Addition of melanoma spheroids and vascularization.	Primary human microvascular endothelial cells (HMVEC), fibroblasts and keratinocytes.	Human melanoma cell lines A375, Malme3M, RPMI-7951 and SK-MEL-28	-	-	Drug testing.	Low throughput. Model takes 5 weeks to fully generate.	[28]
Immune-reactive Planar Human Skin Constructs	Addition of activated CD4+ T cells	Primary keratinocytes.	-	Allogeneic activated CD4+ T cells and in vitro polarized Th1 and Th17.	Decellularized de-epidermized dermis.	Skin inflammation and psoriasis.	Dermis does not contain fibroblasts or other skin cell types, which could also play a role in psoriatic pathology.	[45]
	Addition of MUTZ3-derived Langerhans cells	Primary keratinocytes and fibroblasts.	-	MUTZ-3 progenitor cell line differentiated into Langerhans cells (LCs).	Rat Tail Collagen I.	Allergy; irritant/allergen exposure.	Model does not account for the interaction with other skin cell types such as melanocytes or adipocytes.	[44]
	Addition of activated CD4+ T cells.	Primary keratinocytes and fibroblasts.	-	Allogeneic CD4+ T cells from psoriatic patients and in vitro polarized Th1 and Th17 T cells.	Collagen I.	Psoriasis. Drug screening platform for psoriasis.	Skin cells and immune cells are not donor matched, which may yield unexpected immune responses	[13]
	Filaggrin-deficient cells with activated CD4+ T cells	Primary keratinocytes and fibroblasts.	-	Allogeneic activated CD4+ T cells and in vitro polarized Th1 and Th17.	Bovine collagen I.	Atopic dermatitis.	Immune cell donors coud be dermatitis patients or have allergies that could affect the outcome of the study.	[12]
	3D bioprinted constrcuts with macrophages.	Primary fibroblasts and keratinocytes isolated from human skin donors	-	Matched CD14+ monocytes differentiated into M1 and M2 macrophages.	Bioink with nanofibrilar cellulose, sodium alginate, fibrinogen, mannitol and HEPES.	Chronic skin irritation seen in atopic dermatitis	Model lacks other important cell types such as melanocytes and proper vascularization.	[46]
	Wounded 3D bioprinted constructs containing macrophages and endothelial cells	Primary fibroblasts and keratinocytes. Human umbilical vascular endothelial cells (HUVECs).	-	KG-1 macrophage cell line.	Collagen I for the human planar skin construct. Plasma-derived fibrinogen-containing factor XIII, fibronectin, thrombin for the bioink.	Cutaneous wound healing.	Model lacks other important cell types such as melanocytes.	[47]
Melanoma on Immune-Competent Planar Human Skin Constructs Containing	Addition of melanoma cells.	Primary keratinocytes, melanocytes and fibroblasts.	Human melanoma cell lines SK-MEL-28, A375, COLO829, G361, MeWo and RPMI-7051.	-	Rat Tail Collagen I mixed with Fibrinogen.	Melanoma progression and invasion. Immune evasion mechanisms.	Model lacks integration of immune cells.	[23,58]
	Addition of melanoma and dendritic cells.	Primary keratinocytes and fibroblasts.	Human melanoma cells BLM, Mel624 and A375.	Immunostimulatory dendritic cells (cDC2s).	De-epidermized, decellularized dermis.	Melanoma immune-evasion mechanisms. Potential immunotherapy testing.	Model lacks blood vessels and lymphatics. Unable to reproduce angiogenesis and leukocyte extravasation.	[59]
	Addition of melanoma and T cells.	Primary keratinocytes and fibroblasts (aged and young).	Human melanoma cell lines 1205Lu and WM3918 and patient-derived melanoma cells.	Autologous CD4+ T cells.	Rat Tail Collagen I.	Effects of young and aged fibroblasts on melanoma progression and immune interaction.	Model lacks blood vessels and lymphatics. Unable to reproduce angiogenesis and leukocyte extravasation.	[60]
Melanoma-on-a-chip	Microfluidic device connecting keratinocytes, fibroblasts and melanoma cells.	Primary keratinocytes and fibroblasts.	Primary melanoma cell line WM-115.	-	Collagen	Crosstalk between melanoma cells, fibroblasts and keratinocytes.	Model lacks layered skin architecture and other cell types that may be involved in the crosstalk with cancer cells.	[31]
Immune-competent skin-on-chip	2-layered skin-on-chip containing dendritic cells	Immortalized human keratinocytes (HaCaT).	-	Leukemic monocyte lymphoma cell line U937	-	Skin allergy, skin sensitization, dermatitis.	Model lacks dermis, which homes immune cells that could modify immune responses.	[50]
	Vascularized skin-on-chip.	Primary or immortalized keratinocytes (HaCaT), primary fibroblasts and HUVECs.	-	Human promyelocytic leukemia cell line HL-60	Collagen I.	Neutrophil migration to inflammation sites.	Model lacks skin-resident immune cells that could also affect the immune response against UV and skin irritants.	[51]
	2-layered skin-on-chip containing HUVECs and T cells	Immortalized human keratinocytes (HaCaT), and HUVECs.	-	T cells isolated from peripheral blood samples.	Rat Tail Collagen I.	T cell migration in response to inflammation. Platform for drug testing.	Model lacks fibroblasts in the dermal component, which could affect migration of T cells towards the epidermis.	[14]
Immune-competent melanoma-on-chip	Addition of patient-derived melanoma spheroids.	-	Patient-derived melanoma spheoroids.	Autologous myelooid and lymphoid populations frombiopsy tissue.	Rat Tail Collagen I.	Immunotherapy testing.	Model lacks healthy skin components and layered architecture. They do not take into account the recruitment of other cell types to the TME.	[9]
	Addition of patient-derived melanoma spheroids.	-	Patient-derived melanoma spheoroids.	Immune cells from tissue biopsy.	Rat Tail Collagen I.	Immunotherapy target discovery.	Model lacks healthy skin components and layered architecture. They do not take into account the recruitment of other cell types to the TME.	[61]
	Addition of endothelial cells stimulated with melanoma supernatants and perfused with whole blood	HUVECs.	Human melanoma cells BLM.	Whole blood	Gelatin.	Inflammation induced by the Interaction between melanoma ECM and endothelial cells.	Model lacks healthy skin cells and layered architecture.	[62]
	Vascular network with circulated melanoma and immune cells	-	Human GFP expressing melanoma A-375 and A-375 MA2	Human neutrophils from fresh human blood.	Fibrin.	Intravascular tumor-neutrophil interactions. Cancer cell arrest in vessels and extravassation — metastasis.	Model does not represent skin architecture.	[64]
